# Comparison of adjunctive therapy with metformin and acarbose in patients with Type-1 diabetes mellitus

**DOI:** 10.12669/pjms.333.12669

**Published:** 2017

**Authors:** Amir Ziaee, Neda Esmailzadehha, Maryam Honardoost

**Affiliations:** 1 Amir Ziaee, Professor of Endocrinology, Padiatric Growth and Development Research Center, Institute of Endocrinology and Metabolism, ran University of Medical Sciences, Tehran, Iran; 2 Neda Esmailzadehha, General Practitioners, Metabolic Diseases Research Center, Qazvin University of Medical Sciences, Qazvin, Iran; 3 Maryam Honardoost, Assistant Professor of Molecular Medicine, Endocrine Research Center, Institute of Endocrinology and Metabolism, ran University of Medical Sciences, Tehran, Iran

**Keywords:** Acarbose, Metformin, Type-1 diabetes mellitus

## Abstract

**Objective:**

All the aforementioned data have stimulated interest in studying other potential therapies for T1DM including noninsulin pharmacological therapies. The present study attempts to investigate the effect of adjunctive therapy with metformin and acarbose in patients with Type-1 diabetes mellitus.

**Method:**

In a single-center, placebo-controlled study (IRCT201102165844N1) we compared the results of two clinical trials conducted in two different time periods on 40 patients with Type-1 diabetes mellitus. In the first section, metformin was given to the subjects. After six months, metformin was replaced with acarbose in the therapeutic regimen. In both studies, subjects were checked for their BMI, FBS, HbA1C, TGs, Cholesterol, LDL, HDL, 2hpp, unit of NPH and regular insulin variations.

**Results:**

Placebo-controlled evaluation of selected factors has showna significant decrease in FBS and TG levels in the metformin group during follow up but acarbose group has shown substantial influence on two hour post prandial (2hpp) and regular insulin intake decline. Moreover, Comparison differences after intervention between two test groups has shown that metformin has had superior impact on FBS and HbA1C decline in patients. Nonetheless, acarbose treatment had noteworthy influence on 2hpp, TGs, Cholesterol, LDL, and regular insulin intake control.

**Conclusion:**

The results of this experiment demonstrate that the addition of acarbose or metformin to patients with Type-1 diabetes mellitus who are controlled with insulin is commonly well tolerated and help to improve metabolic control in patients.

## INTRODUCTION

Type-1 diabetes mellitus (T1DM), is a complex chronic condition^1^-^3^ that results from autoimmune-mediated beta cell destruction and leads to an absolute insulin deficiency.^4^ Insulin is the core treatment of T1DM. Achieving stable blood glucose levels and adequate hemoglobin A1c (HbA1c)and preventing the development of micro vascular and macro vascular complications are the main goals of treatment in T1DM.^5^,^6^ The Diabetes Control and Complications Trial has shown that intensive insulin regimens to reduce glucose to near-normal levels are associated with delays in incidence and progression of diabetes-related complications.^3^,^4^,^7^

Although beneficial, insulin therapy has some shortcomings. Insulin resistance may develop in some patients. Intensive insulin therapy is also associated with increased risk of hypoglycemia, and weight gain.^3^ Thus the goals of good glycemic control are often not achievable.^8^ Physiologic changes in insulin sensitivity during growth and pubertal development also results in insulin resistance in both adult and youth with T1DM.^9^ In addition to glycemic control, patients with T1DM do not meet the targets of metabolic control for BMI, blood pressure, low-density lipoprotein (LDL)-cholesterol, and triglycerides.^2^,^10^,^11^

All the aforementioned data have stimulated interest in studying other potential therapiesfor T1DM including noninsulin pharmacological therapies. Since Type-2 diabetes mellitus (T2DM) is mainly the result of insulin resistance,^12^,^13^ currently approved medications for the treatment of Type-2 diabetes focus on reducing insulin resistance and preventing weight gain by different mechanisms of action.^14^ Acarbose (Alpha-Glucosidase Inhibitor) and metformin are of T2DM medications. Acarbose inhibits digestion into monosaccharides and offsetting post-prandial glucose rise while metformin inhibits hepatic glucose production and improves peripheral insulin sensitivity.^2^,^12^,^15^,^16^ The effectiveness of acarbose and metformin in patients with T1DM has been evaluated in previous studies separately but studies that focus on comparing the effect of these two drugs are limited. The aim of this study was to compare adjunctive therapy with metformin and acarbose in patients with Type-1 diabetes mellitus.

## METHODS

The current study compared the results of twoplacebo-controlled clinical trials conducted in two different time periods on 40 patients with Type-1 diabetes. Both clinical trials were approved by the Ethics Committee of Institute of Endocrinology and Metabolism, Iran University of Medical Sciences (IRCT201102165844N1). Patients participating in these clinical trials were selected from the ones who referred to the endocrinologist’s office and enrolled after signing the written informed consent. The inclusion criteria were: age 15-25 years, puberty stage of 2-5 Tanner, at least three years history of diabetes, HbA1C 7-11 (within the recent six months). The exclusion criteria were: diabetic nephropathy (albumin excretion over 300 mg/dL), proliferative retinopathy, liver or renal failure and any severe illness, eating disorders and a history of recurrent diabetic ketoacidosis (more than two times within the last year leading to reduction of the level of consciousness and hospitalization). The patient groups went through two treatment interventions.

In the first study, metformin was added to the therapeutic regimen of the subjects and results were compared with that of the placebo group. The initial dose of metformin was 500 mg/day and gradually increased from the first week, in a way that the maximum doses in the subjects under 50 kg, 50-70 kg and over 75 kg were increased to 1000, 1500 and 2000 mg, respectively. Subjects were advised to take the medicine with food to reduce digestive complications. The study period was three months and subjects were examined every month and monitored by telephone every week.

Six month after the first study and during the second study, acarbose was added to the therapeutic regimen of the subjects and the results were compared to those of the placebo group. Subjects received acarbose 25 mg three times a day for two weeks and then 50 mg three times a day for 10 weeks. The regimen was administered as tablets before each meal. The subjects were advised to monitor their blood sugar by glucometer and use glucose powder whenever symptoms of hypoglycemia i.e. lethargy, sweating and heart palpitations develop; if their blood glucose was less than 50 mg/dL, they were advised to refer to the hospital. Subjects were examined every month and monitored by telephone every week. In both studies, subjects were advised to check their fasting blood sugar (FBS), two-hour postprandial plasma glucose (2hpp) after each meal and add a unit of NPH insulin (Neutral Protamine Hagedorn) every night for each extra 50 mg/dLof FBS higher than 150 mg/dL; and add two units of regular insulin to the same turn for each 2hpphigher than 200 mg/dL. In addition, they were advised to record the frequencies and the time of hypoglycemia and the interval from the previous meal. They were also advised to refer to the hospital in case of symptomatic hypoglycemia and to stay in touch with the plan executor to match all insulin doses. The adverse effects caused by oral medication (bloating, stomachache, diarrhea and hypoglycemia) were recorded.

The body weight of the subjects were monitored monthly using a digital scale with accuracy of 0.1 kg, the height was measured by the tape meter while their heels and shoulders were attached to the wall; the blood pressure was measured using a Richter mercury sphygmomanometer with appropriate brachial cuff. The measurements were conducted by the same person. Body mass index (BMI) was measured based on the equation: weight (in kg) divided by the square of height (in meter). Also, the mean of insulin dose was measured based on insulin dosage and the mean of FBS and 2hpp during the last seven days before monthly examination.

The blood glucose was measured in home by the subjects using Accuchek glucometer device. Finally, HbA1c was measured using Jame-eh kit through column chromatography; the levels of triglyceride, cholesterol, high density and low density lipoproteins (HDL and LDL) were measured using Pars kit (Iran) by GHOD-PAP and Hyper-G; the level of LDL was measured using Pars Azmoon kit (Iran) by enzymatic method in the beginning and at the end of the study.

Data were presented as mean ± SD. The mean of evaluated variables were compared before and after the treatment, using paired T-test. The difference between the mean of variables before and after the treatment were calculated and compared with the results of the paired T-test for the two types of treatment.

## RESULTS

Mean age was 19.31±1.25 in the beginning of the first study. Of 20 patients, 10 were male. None of the patients were excluded or missed to follow up in both studies. The comparison of laboratory characteristics and insulin intake before and after adjunctive therapy with metformin and acarbose has been shown in Table-I. After treatment with metformin, mean FBS and TG was significantly decreased; but only mean two hour post prandial and regular insulin intake were significantly decreased after treatment with acarbose.

**Table-I:** Comparison of laboratory characteristics and insulin intake before and after adjunctive therapy with metformin and acarbose in T1DM patients.

	*Before*	*After*	*P-value*
*Metformin*			

BMI	23.21±1.40	22.80±1.80	0.410
FBS	168.80±19.90	113.56±14.90	<0.001
2 h pp	198.70±18.5	182.70±16.30	0.006
HbA1C	8.36±0.80	8.02±0.63	0.143
TGs	96.90±14.0	87.94±13.45	0.045
Cholesterol	135.75±24.10	131.81±22.65	0.597
HDL-C	35.12±9.80	36.28±9.60	0.707
LDL-C	81.00±12.66	79.25±13.90	0.679
Insulin (NPH)	33.40±8.20	31.60±8.10	0.489
Insulin (Regular)	18.5±7.60	17.60±6.80	0.695

*Acarbose*			

BMI	23.96±1.70	23.70±1.40	0.600
FBS	130.30±18.90	120.10±18.70	0.094
2 h pp	180.20±22.70	154.80±18.70	<0.001
HbA1C	7.80±0.45	7.60±0.40	0.145
TGs	166.40±33.60	148.80±30.95	0.093
Cholesterol	172.80±22.90	162.30±21.10	0.139
HDL-C	38.60±5.70	38.96±5.80	0.844
LDL-C	101.0±21.40	93.56±20.60	0.269
Insulin (NPH)	35.40±8.40	33.80±7.30	0.524
Insulin (Regular)	20.20±6.30	15.80±5.25	0.021

BMI: body mass index; FBS: fasting blood sugar; 2h pp: 2-hour post-prandial blood sugar; HbA1C: hemoglobin A1c; TG: triglycerides; HDL: high-density lipoprotein cholesterol; LDL: low-density lipoprotein cholesterol; Insulin (NPH): Isophane insulin.

Comparison of mean difference after intervention between two groups has been shown in Table-II. The mean decrease in FBS and HbA1C after treatment with metformin was significantly higher than treatment with acarbose. The mean decrease in 2hpp, TGs, Cholesterol, LDL, and regular insulin intake after treatment with acarbose was significantly higher than treatment with metformin.

**Table-II:** Comparison of mean difference after intervention between two groups.

	*Mean difference after intervention*		*P-value*

	*Metformin*	*Acarbose*	
BMI	0.41±0.31	0.25±0.46	0.204
FBS	-55.24±14.3	-10.20±8.43	<0.001
2 h pp	-16.1±4.4	-25.30±12.46	0.003
HbA1C	-0.34±0.21	-0.288±0.19	<0.001
TGs	-8.96±6.4	-17.50±7.90	<0.001
Cholesterol	-3.94±1.1	-10.50±8.70	0.002
HDL-C	-1.14±0.42	-0.40±1.90	0.097
LDL-C	-1.75±2.43	-7.44±9.20	0.011
Insulin (NPH)	-1.8±0.74	-1.56±1.80	0.584
Insulin (Regular)	-0.9±1.4	-4.30±2.05	<0.001

BMI: body mass index; FBS: fasting blood sugar; 2h pp: 2-hour post-prandial blood sugar; HbA1C: hemoglobin A1c; TG: triglycerides; HDL: high-density lipoprotein cholesterol; LDL: low-density lipoprotein cholesterol; Insulin (NPH): Isophane insulin

## DISCUSSION

In order to provides an efficacious and safe alternative for glycemic improvement in Type-1 patients mellitus, our test group received metformin firstly and then acarbose during the trial. It has been shown that acarbose/metformin is well-tolerated in T1DM patients. Based on our result, a significant decrease in FBS and TG levels was observed in the metformin group during follow-up but acarbose has shown substantial influence on 2 h pp and regular insulin intake decline in treated patients. Variation in HbA1C, Cholesterol, LDL, HDL level, BMI and NPH insulin was not significant in both groups when compared with placebo. Although not statistically significant, there was a trend toward reductions in HbA1C in those treated with both drug (P =0.143).

Our metformin group result was similar to some reported studies.^4^,^5^,^7^ For instance, a meaningful decrease in TG and PPG or a significant reduction in fasting blood glucose level have been reported in metformin group compared with placebo based on different studies.^4^,^5^ However, there are some controversial aspects about metformin consequences on regular insulin intake, HbA1C or BMI index. Although several investigators believe that there are beneficial effects on HbA1c level,^4^,^7^ others claim that there is no significant results for those taking metformin and our result analysis put us in the last set.^17^

Alternatively, about acarbose function in diabetic patients, although our results have indicated that the use of medication was associated with significant drop in daily insulin requirements and 2hpp levels, Hollander and colleagues demonstrated that acarbose was associated with significant reductions in HbA1c and there is no significant changes in total daily insulin doses or body weight.^18^ Nevertheless, Juntti-Berggren, found that acarbose did produce significantly lower insulin requirements^19^ which has the same outcomes as our project. Some of former publications showed just improvement in post-prandial glucose levels but no impact on HbA1c^20^ or reported that acarbose did decrease HbA1c, FBS and 2-hpp level considerably.^4^ It seems that these contradictory outcomes occur because of different approach in each trial.

In future, for better understanding of medication paybacks we have compared the mean difference of all evaluated factors after intervention between two test groups (Table-II). Interestingly, in our data, while metformin has shown more decrease in FBS and Hb A1C level, acarbose has had better effect on improved postprandial glucose level and TG, cholesterol, LDL and regular insulin doses reductions.

Compared with insulin monotherapy, adjunctive therapy with acarbose/metformin has greater antihyperglycemic ability, brings proportionally more T1DM patients to HbA1c goal, and further reduces lipid consumptions. Hence, using insulin sensitive pharmacological drugs may have positive properties on Type-1 diabetes mellitus treatment and improvement of metabolic control.

### Author’s Contribution

**AZ:** Conception & design, acquisition, interpretation of data, final approval of the version to be published.

**NE and MH:** Acquisition of data, statistical analysis, drafting the article, final approval of the version to be published.

**AZ, NE and MH:** Accountable for all aspects of the work in ensuring that questions related to the accuracy or integrity of any part of the work are appropriately investigated and resolved.
